# Characterization of cefotaxime resistant *Escherichia coli* isolated from broiler farms in Ecuador

**DOI:** 10.1371/journal.pone.0207567

**Published:** 2019-04-05

**Authors:** Christian Vinueza-Burgos, David Ortega-Paredes, Cristian Narváez, Lieven De Zutter, Jeannete Zurita

**Affiliations:** 1 Unidad de Investigación de Enfermedades Transmitidas por Alimentos y Resistencias a los Antibióticos, Facultad de Medicina Veterinaria y Zootecnia, Universidad Central del Ecuador, Quito, Ecuador; 2 Unidad de Investigaciones en Biomedicina, Zurita&Zurita Laboratorios, Quito, Ecuador; 3 Department of Veterinary Public Health and Food Safety, Faculty of Veterinary, Ghent University, Merelbeke, Belgium; University Hospital Basel, SWITZERLAND

## Abstract

Antimicrobial resistance (AR) is a worldwide concern. Up to a 160% increase in antibiotic usage in food animals is expected in Latin American countries. The poultry industry is an increasingly important segment of food production and contributor to AR. The objective of this study was to evaluate the prevalence, AR patterns and the characterization of relevant resistance genes in Extended Spectrum β-lactamases (ESBL) and AmpC-producing *E*. *coli* from large poultry farms in Ecuador. Sampling was performed from June 2013 to July 2014 in 6 slaughterhouses that slaughter broilers from 115 farms totaling 384 flocks. Each sample of collected caeca was streaked onto TBX agar supplemented with cefotaxime (3 mg/l). In total, 176 isolates were analyzed for AR patterns by the disk diffusion method and for *bla*_CTX-M_, *bla*_TEM_, *bla*_CMY_, *bla*_SHV_, bla_*KPC*_, and *mcr-1* by PCR and sequencing. ESBL and AmpC *E*. *coli* were found in 362 flocks (94.3%) from 112 farms (97.4%). We found that 98.3% of the cefotaxime-resistant isolates were multi-resistant to antibiotics. Low resistance was observed for ertapenem and nitrofurantoin. The most prevalent ESBL genes were the ones belonging to the *bla*_*CTX-M*_ group (90.9%), specifically the *bla*_*CTX-M-65*_, *bla*_*CTX-M-55*_ and *bla*_*CTX-M-3*_ alleles. Most of the AmpC strains presented the *bla*_*CMY-2*_ gene. Three isolates showed the *mcr-1* gene. Poultry production systems represent a hotspot for AR in Ecuador, possibly mediated by the extensive use of antibiotics. Monitoring this sector in national and regional plans of AR surveillance should therefore be considered.

## Introduction

Antimicrobial resistance (AR) is a worldwide concern. It is expected that deaths linked to AR could rise from 700,000 to 10 million deaths per year by 2050[1]. On the other hand, In developed countries, an estimated 23,000 (United States) to 25,000 (Europe) deaths are attributable to resistant pathogens each year [2].

In terms of economic loses, AR could cause a global loss of production as high as 100 trillion dollars which represents a huge impact on the economy of all countries, especially developing countries [1]. This problem will not only result in increased illnesses, disabilities and deaths but also puts at risk the achievement of Sustainable Development Goals for the next 30 years [3,4].

It has been observed that AR in relevant pathogens has increased in recent years [5,6]. The causes of this phenomenon are complex and are mainly linked to selective pressures triggered by antibiotic usage [7]. Inappropriate prescription of antimicrobials, unauthorized sale of antibiotics and the extensive use of these drugs in animal production are mayor factors that contribute to this problem [8,9]. For example, in 2010, more than 63,000 tons of antibiotics were used in livestock [10]. Moreover, it has been projected that by 2030 the use of antibiotics in livestock will double in countries such as Brazil, Russia, India and South Africa. Furthermore, up to a 160% increase in antibiotic usage in food animals is expected in Latin American countries [11].

Worldwide, the poultry industry is an increasingly important segment of food production. In fact, it is expected that by 2025, poultry will be the most important sector of meat production [11,12]. Widespread use of antibiotics in the poultry industry impacts the AR problem. This issue is especially relevant in developing countries where antimicrobials are not only used therapeutically but also prophylactically and as growth promoters [13,14].

Among the antibiotics used in livestock production, some are listed in the WHO list of critically important antimicrobials for human medicine. This group includes third generation cephalosporines, carbapenems and colistin, all of which are categorized as highest priority [15]. Additionally, extended-spectrum β-lactamases (ESBL)- and AmpC-producing *E*. *coli*, and carbapenem-resistant *E*. *coli* are listed as high priority organisms for which new antibiotics are urgently needed [16].

*E*. *coli* harboring resistance determinants originating in the poultry industry are therefore of great epidemiological interest because they can serve as reservoirs of resistance genes that can be transferred to human pathogens [17]. A relationship between resistant strains of *E*. *coli* from poultry and those found in humans has been suggested in several studies [18–20]. However, information about resistant *E*. *coli* in industrial poultry has been poorly studied in Latin America. The objective of this study was to evaluate the prevalence and AR patterns of and to characterize relevant resistance genes in ESBL and AmpC *E*. *coli* from large poultry farms in Ecuador.

## Material and methods

### Study design and sampling

Pichincha, the province where Quito, the capital city of Ecuador is located, was selected for the collection of samples since 36% of the total Ecuadorian broiler production is located in this and surrounding provinces [21]. Eight large slaughterhouses are located in Pichincha [21] and all were asked to participate in the study. Sampling was performed in the 6 participating slaughterhouses which slaughter broilers from 115 farms with a median capacity of 100.000 broilers. These farms deliver broilers to be consumed mainly at Pichincha province. From June 2013 to July 2014, a total of 384 flocks (birds coming from one house and slaughtered on the same day) were sampled. All sampled flocks from the same farm originated from different houses or birds reared during different periods in the same house.

In Ecuador, commercial broiler farm management includes the total depopulation of houses and removal of the litter after every flock, cleaning and disinfection of the house followed by a dormant period of 8 to 15 days. All sampled flocks were commercially reared and slaughtered at the age of 6 to 7 weeks. From each batch, caeca from 25 randomly selected chickens were collected, and transported in an ice box within 1 hour to the laboratory for bacteriological analysis.

### Isolation and identification of ESBL/AmpC *E*. *coli*

Caeca from each flock were immersed in 98% ethanol to eliminate surface bacteria present due to human handling. After evaporation of the ethanol, approximately 1 g of fecal content from each of the 25 samples was collected and pooled in a sterile plastic bag. The pooled sample was homogenized by hand for 1 minute.

Each sample was streaked onto chromogenic Tryptone Bile X-Glucuronide (TBX) agar (BioRad) supplemented with cefotaxime (3 mg/l) [22]. Positive plates where considered when at least one typical colony could be selected (when possible, two sample colonies were selected) and confirmed to be *E*. *coli* using Triple Sugar Iron agar (Difco, BD) and by PCR [23]. From this medium, one loopful was used to extract DNA by the boiling method. Another loopful was used to subculture the isolate in trypticase soy broth (Difco, BD) and stored with glycerol (60%) at -80°C. All cefotaxime resistant *E*. *coli* isolates were further examined for the presence of ESBL using ceftazidime, ceftazidime/clavulanate, cefotaxime, cefotaxime/clavulanate disks [24] and for the AmpC phenotype using boronic acid, ceftazidime and cefepime disks [25].

### Antimicrobial resistance and PCR screening

When detected, one isolate with ESBL phenotype and/or one isolate with AmpC phenotype from each farm were selected for analysis by the Kirby Bauer method. AR profiles were evaluated using clinical breakpoint values from the Clinical and Laboratory Standards Institute (CLSI,2018) [24]. The following antibiotics were evaluated: trimethoprim-sulfamethoxazole, nalidixic acid, ciprofloxacin, gentamicin, kanamycin, streptomycin, tetracycline, chloramphenicol, fosfomycin, tetracycline, doxycycline, ceftazidime and ertapenem. *E*. *coli* ATCC 25922 was used as a quality control strain.

Selected isolates for AR testing were studied by PCR to identify *bla*_CTX-M_, *bla*_TEM_, *bla*_CMY_, *bla*_SHV_ and bla_*KPC*_. PCR conditions and primers were those described by [26] for *bla*_CTX-M_, [27] for *bla*_TEM_, [28] for *bla*_CMY_, [29] for *bla*_SHV_ and [30] for bla_*KPC*_. Sub-families of *bla*_CTX-M_ genes were identified with PCR protocols described by [31] for *bla*_CTX-M-1_, [32] for *bla*_CTX-M-2_, [33] for *bla*_CTX-M-8_, [34] for *bla*_CTX-M-9_ and [35] for *bla*_CTX-M-14-like_. Additionally, Isolates were tested by PCR for the presence of *mcr*-1 [36] and *mcr*-2 [37] plasmid genes. Amplification products were confirmed by gel electrophoresis using a 1% agarose gel. All PCR products were purified and sequenced at Macrogen Inc. (Seoul, South Korea). Obtained sequences were aligned against reference sequences with the online tool ResFinder 2.1 with an identity threshold of 100% [38]. MIC values for ampicillin, piperacillin-tazobactam, cefoxitin, ceftazidime, ceftriaxone, cefepime, doripenem, ertapenem, imipenem, meropenem, amikacin, gentamicin, ciprofloxacin, tigecycline and colistin were obtained on *mcr* positive isolates using the Vitek 2 system with the AST-N272 card (Biomérieux, Marcy-l'Étoile, France). MIC values for colistin were confirmed by microdilution using GNX2F plates (Thermo Scientific,West Palm Beach, USA) The results were evaluated using the breakpoints recommended by CLSI (2018) [24].

### Genetic characterization

Fingerprint characterization was performed in selected isolates for AR testing by repetitive element palindromic PCR (REP-PCR) analysis [39]. Bands were analyzed using BioNumerics software V.7.6 (Applied Maths, Sint-Martems-Latem, Belgium). Fragments between 200 bp and 1500 bp in size were included in the analysis. The unweighted pair group method using the arithmetic averages algorithm (UPGMA) with a 1.5% tolerance was used to construct a dendrogram. Patterns with more than one isolate were arbitrarily numbered starting from I and ordered from the most frequent one.

### Statistical analysis

To determine the prevalence of *E*. *coli* ESBL at the farm level, a farm was considered positive when at least one of the sampled batches was positive. Farms were assumed to be independent.

Differences in antibiotic resistances between ESBL *E*. *coli* and AmpC *E*. *coli* were calculated by the chi-square test. Proportions were considered significantly different when the *P* value was below 0.05.

## Results

### Prevalence of ESBL and AmpC *E*. *coli* isolates at poultry farms

ESBL and/or AmpC *E*. *coli* were found in 362/384 flocks (94.3%; CI95%: 93,39% - 95,26%). In total, 112/115 farms (97.4%; CI95%: 96.37% - 98.94%) delivered a positive result at least once.

From all positive flocks, 62 (17.1%) delivered a combination of ESBL and AmpC isolates while 223 (61.6%) and 51 (14.1%) flocks had exclusively the ESBL or AmpC phenotypes, respectively. Only one colony could be isolated from 26 flocks, 21 (5.8%) and 3 (0.01%) showing the ESBL and AMPC phenotypes respectively (colonies from 2 flocks could not be recuperated for phenotypic testing).

For AR tests, 110 *E*. *coli* ESBL and 66 *E*. *coli* AmpC isolates were selected for further analysis.

### Antimicrobial resistance patterns

Antimicrobial susceptibility testing grouped ESBL and AmpC *E*. *coli* isolates in 26 patterns that showed resistances to between 2 and 7 antibiotic families tested. Resistance patterns to at least 3 antibiotic families (multi-resistant isolates) were present in 98.3% of all tested isolates. Moreover, 92.1% of isolates presented resistance to between 4 and 7 antibiotic families. Pattern number 3 was the most common one for ESBL and AmpC isolates with resistance to all tested groups of antibiotics with the exception of nitrofurantoin (Table 1), while 9.7% of tested *E*. *coli* isolates presented resistance to all tested groups of antibiotics.

**Table 1:** Antibiotic resistance patterns of ESBL/AmpC *E*. *coli* isolated from poultry farms.

Pattern	Resistance Pattern number	No. of antibiotic groups	No. of ESBL isolates (n = 110)	No. of AmpC isolates (n = 66)	Total isolates (%) (n = 176)
BQTAPNS	1	7	8	9	17 (9.7)
BQTANS	2	6	2	2	4 (2.3)
BQTAPS	3	6	42	19	61 (34.7)
BQTAPN	4	6	1	2	3 (1.7)
BQTPNS	5	6	1	1	2 (1.1)
BQTAS	6	5	9	6	15 (8.5)
BQTAN	7	5	1	1	2 (1.1)
BQTNS	8	5	1		1 (0.6)
QTAPS	9	5		4	4 (2.3)
BQTAP	10	5	6	3	9 (5.1)
BTAPS	11	5	1	2	3 (1.7)
BQAPS	12	5	4	3	7 (4)
BAPNS	13	5	1		1 (0.6)
BQTPS	14	5	2	1	3 (1.7)
BQTA	15	4	12	1	13 (7.4)
BQTS	16	4		2	2 (1.1)
BQAN	17	4		1	1 (0.6)
BAPS	18	4	2	1	3 (1.7)
BQPS	19	4	1	1	2 (1.1)
BQAP	20	4	3	2	5 (2.8)
BQTP	21	4	1	1	2 (1.1)
BTPS	22	4	2		2 (1.1)
BQT	23	3	5	1	6 (3.4)
BQA	24	3	1	1	2 (1.1)
BTA	25	3	1	2	3 (1.7)
BQ	26	2	3		3 (1.7)

B, Beta-lactam; Q, quinolone; T, tetracycline; A, aminoglycoside; P, phenicols; N, nitrofuran; S, sulfonamide.

Number of isolates and AR rates for ESBL and AmpC *E*. *coli* isolates are shown in Table 2. Low resistance rates were observed for ertapenem followed by nitrofurantoin. For the remaining antibiotics, resistance rates ranged from 29,1% to 93.9%. Antibiotics for which significant differences were observed between ESBL and AmpC isolates were ceftazidime, kanamycin and gentamicin. Resistance to ceftazidime and kanamycin was more frequent in AmpC isolates while in ESBL isolates resistace to gentamicin was more frequent (Table 2).

**Table 2:** Number of ESBL/AmpC *E*. *coli* isolates resistant to each tested antibiotic.

Antibiotic group	Antibiotic	No. of ESBL isolates (%) (n = 110)	No. of AmpC isolates (%) (n = 66)
Beta-lactams	*Ceftazidime	32 (29.1)	62 (93.9)
	Ertapenem	0 (0)	1 (1.5)
Quinolones	Nalidixic acid	103 (93.6)	61 (92.4)
	Ciprofloxacin	80 (72.7)	47 (71.2)
Tetracyclines	Tetracycline	95 (86.4)	27 (86.4)
	Doxycycline	82 (74.5)	54 (81.8)
Aminoglycosides	Streptomycin	91 (82.7)	56 (84.8)
	*Kanamycin	44 (40)	42 (63.6)
	*Gentamicin	52 (47.3)	18 (27.3)
Sulfonamide	Sulfamethoxazole + trimethoprim	76 (69.1)	51 (77.3)
Phenicol	Chloramphenicol	75 (68.2)	49 (74.2)
Nitrofuran	Nitrofurantoin	15 (13.6)	16 (24.2)

* ESBL and AmpC isolates showed significantly different rates by chi-square test (p<0.05) for these antibiotics.

Four isolates from the ESBL group (n = 110; 1CT86A, 1CT109B, 1CT136A and 1CT160A) and 2 isolates from the AmpC group (n = 66; 1CT22A and 1CT188B) were positive for the *mcr-*1 gene. The six isolates originated in different farms. MIC values of these isolates are shown in Table 3. All these isolates presented resistance to ceftriaxone, and colistin. Resistance to doripenem, imipenem, amikacin and tigecycline was not identified in any isolate.

**Table 3:** MIC values of *mcr*-1 positive isolates.

Isolate	Isolation date (mm/yyyy)	ESBL/AmpC genes	AMP	TPZ	FOX	CAZ	CRO	FEP	DOR	ERT	IMI	MER	AK	GEN	CIP	TIG	COL
1CT22A	08/2013	*bla*_CMY-2_	**≥ 32**	**≥ 128**	**≥ 64**	**16**	**16**	≤ 1	≤0,12	≤0,5	≤0,25	≤0,25	≤2	**≥ 16**	**≥ 4**	≤0,5	**8**
1CT86A	12/2013	*bla*_CTX-M-2_	**≥ 32**	≤ 4	**16**	4	**≥ 64**	**≥ 64**	≤0,12	≤0,5	≤0,25	≤0,25	≤2	**≥ 16**	**≥ 4**	≤0,5	**8**
1CT109B	01/2014	*bla*_CTX-M-14_	**≥ 32**	≤ 4	8	≤ 1	**≥ 64**	2	≤0,12	≤0,5	≤0,25	≤0,25	≤2	**≥ 16**	**≥ 4**	≤0,5	**4**
1CT136A	03/2014	*bla*_CTX-M-65_	**16**	≤ 4	≤ 4	≤ 1	**16**	≤ 1	≤0,12	≤0,5	≤0,25	≤0,25	≤2	**≥ 16**	**≥ 4**	≤0,5	**8**
1CT160A	04/2014	*bla*_CTX-M-65_	**≥ 32**	≤ 4	**16**	**32**	**≥ 64**	4	0,5	**1**	≤0,25	**4**	4	**≥ 16**	**≥ 4**	≤0,5	**≥ 16**
1CT188B	06/2014	*bla*_CMY-2_	**16**	≤ 4	**≥ 64**	4	**8**	≤ 1	≤0,12	≤0,5	≤0,25	≤0,25	≤2	1	≤0,25	≤0,5	**8**

AMP: ampicillin; TPZ: piperacillin-tazobactam; FOX: cefoxitin; CAZ: ceftazidime; CRO: ceftriaxone; FEP: cefepime; DOR: doripenem; ERT: ertapenem; IMI: imipenem; MER: meropenem; AK: amikacin; GEN: gentamicin; CIP: ciprofloxacin; TIG: tigecycline; COL: colistin.

Values in bold indicate resistance according to CLSI-2018.

### Genetic characterization

Sequencing of genes in isolates with ESBL phenotype (n = 110) showed that the most prevalent family of genes was *bla*_*CTX-M*_ (90.9%)_._ Forty-eight (43.6%) and 9 (8.2%) *bla*_*CTX-M*_ positive isolates presented the *bla*_*TEM-1A*_ or *bla*_*TEM-1B*_, and *bla*_*SHV-5*_ alleles respectively. Three isolates had the *bla*_*TEM-176*_ gene and 1 isolate presented the *bla*_*SHV-153*_ gene. Three and one isolates presented only *bla*_*SHV-5*_ and *bla*_*SHV-153*_ respectively_._ One and 4 isolates presented only *bla*_TEM-1A_ or *bla*_TEM-1B_ genes respectively (4,5%) which do not hydrolyze cefotaxime. Within isolates that presented genes of *bla*_*CTX-M*_ family (n = 100), *bla*_*CTX-M-65*_, *bla*_*CTX-M-55*_ and *bla*_*CTX-M-3*_ accounted for 77 isolates. One isolate was not positive for any of the studied ESBL genes (Table 4).

**Table 4:** Combination of *bla*_*CTX-M*_, *bla*_*TEM*_ and *bla*_*SHV*_ alleles in ESBL *E*. *coli* isolates.

*bla*_CTX-M_ group (%) (n = 110)	*bla*_CTX-M_ allele (%)	*bla*_TEM_	*bla*_SHV_	No. of isolates
**Group 1** **45.5%** **(n = 50)**	*bla*_CTX-M-1_ (**3.6%)**	-	-	2
		*-*	-	1
		*-*	-	1
	*bla*_CTX-M-3_ (**19.1%)**	-	-	14
		*-*	-	2
		*-*	-	2
		*bla*_TEM-176_	-	2
			*bla*_SHV-5_	1
	*bla*_CTX-M-12_ (**2.7%)**	-	-	1
		-	*bla*_SHV-5_	2
	*bla*_CTX-M-29_ (**0.9%)**	-	-	1
	*bla*_CTX-M-55_ (**17.3%)**	-	-	1
		*-*	-	13
		-	*bla*_SHV-5_	4
		*bla*_TEM-176_	*bla*_SHV-5_	1
	*bla*_CTX-M-123_ (**1.8%)**	*-*	-	2
**Group 2** **7.3%** **(n = 8)**	*bla*_CTX-M-2_ (**7.3%)**	-	-	3
		*-*	-	1
		*-*	-	3
		*-*	*bla*_SHV-5_	1
**Group 8** **0,9%** **(n = 1)**	*bla*_CTX-M-8_ (**0.9%)**	-	-	1
**Group 9** **37,3%** **(n = 41)**	*bla*_CTX-M-14_ (**1.8%)**	-	-	2
	*bla*_CTX-M-21_ (**0.9%)**	*-*	-	1
	*bla*_CTX-M-27_ (**0.9%)**	-	-	1
	*bla*_CTX-M-65_ (**33.6%)**	-	-	15
		-	-	1
		*-*	-	1
		*-*	-	20

Of the 66 AmpC isolates, 7 were PCR negative for *bla*_*cmy*_, 58 were positive for *bla*_*cmy-2*_, and 1 isolate was positive for *bla*_*cmy-46*_. None of the 176 tested isolates were positive for *bla*_*KPC*_ or *mcr*-2.

REP-PCR delivered 121 genotypes (S1 Fig) from which 22 grouped to more than one isolate (Table 5).

**Table 5:** Genotypes of ESBL and AmpC *E*. *coli* with more than one isolate.

Genotypes	No. of isolates		
	ESBL	AmpC	Total
**I**	14		14
**II**	10	1	11
**III**	3	2	5
**IV**	4	1	5
**V**	4		4
**VI**	3		3
**VII**	3		3
**VIII**		3	3
**IX**	2		2
**X**	2		2
**XI**	2		2
**XII**	2		2
**XIII**	2		2
**XIV**	2		2
**XV**	2		2
**XVI**	1	1	2
**XVII**	1	1	2
**XVIII**		2	2
**XIX**		2	2
**XX**		2	2
**XXI**		2	2
**XXII**		2	2

Genotypes II, III, IV, XVI and XVII were present in both ESBL and AmpC isolates. In total, genotypes I and II were the most common ones, with 14 and 11 isolates, respectively. ESBL and AmpC isolates from each genotype originated from different farms.

## Discussion

The aim of this research was to study AR in *E*. *coli* from intensive poultry farming. The sparseness of this kind of data from Latin America makes this study one of the few available reports that demonstrate the extent of ESBL/AmpC *E*. *coli* in commercial poultry in the region [40–42]. Nonetheless, developed countries have also reported both a high prevalence of ESBL *E*. *coli* and the presence of multiresistant isolates from broiler flocks [43,44].

Similar to a previous studie in small-scale poultry farming in Ecuador [45], this research shows high prevalence of ESBL genes (*bla*CTX-M) among cefotaxime-resistant *E*. *coli*. However, a study carried out in Colombia by Castellanos et al. [41] demonstrates a higher prevalence of AmpC genes (*bla*CMY) in cefotaxime-resistant *E*. *coli* isolates from commercial poultry. Differences in the epidemiologic patterns of enteric bacteria isolated from Ecuadorian and Colombian poultry has been reported before and may be attributed to the ecological characteristics (altitude above 2800 m.a.s.l.) of the boundary between these neighboring countries [13].

High AR rates and multi-resistance patterns could be related to the intensive use of antimicrobials in poultry production, which in some cases are not only used as therapeutics but also as prophylactics and growth promotors [11,46]. On the other hand, it has to be considered that the withdrawal of antibiotics from poultry production systems may not result in the diminution of ESBL/AmpC *E*. *coli* since ecological factors could be implicated in the dynamics of AR determinants [47,48].

Increasing antibiotic resistance and the lack of new antibacterial agents have revived interest in old compounds such as nitrofurantoin in clinical practice [49,50]. Despite the renewed importance of nitrofurantoin and the known role of food animals in resistance dissemination, only a few studies include this antibiotic in AR screenings [51,52]. Resistance rates to nitrofurantoin in this study are higher than the ones reported in chicken meat samples (7,9%) in Colombia by Donado-Godoy et al. [52]. Higher resistance to nitrofurantoin in extra-intestinal clinical isolates of *E*. *coli* from chickens has been reported in China [51]. Although nitrofurantoin is not used in the poultry industry in Ecuador, surveillance of this antibiotic should carried out in poultry production systems due to the possibility that the resistance to this antibiotic could increase over time.

Carbapenems are not used in the poultry industry in Ecuador, resulting in a lack of selective pressure by this antimicrobial in poultry production. This observation explains the low prevalence of carbapenem-resistant *E*. *coli* reported in poultry [53]. Concordantly, we only identified one isolate resistant to ertapenem and meropenem, although carbapenem resistance mediated by *bla*_KPC_ has been reported in clinical Enterobacteriaceae in Ecuador [54].

The association of ESBL and AmpC phenotypes with increased prevalence of aminoglycoside resistance has been reported [55]. In our study we identified a significant association of kanamycin resistance with AmpC-producing isolates and gentamicin resistance to ESBL-producing *E*. *coli*. Further genetic characterization should be performed in order to explain whether these ARs are associated with specific genetic environments (the presence of aminoglycosides modifying enzymes), In our study, all selected isolates were resistant to cefotaxime (used for screening), however, AmpC isolates were significantly more resistant to ceftazidime than ESBL isolates. This difference is explained by the enhanced hydrolyzation of ceftazidime by the *bla*_*CMY*_ gene product [56,57].

Several studies throughout the world have reported plasmid-mediated colistin resistance in Enterobacteriaceae pointing to its global emergence [58]. In our study 6 out of 176 isolates (3.4%) were PCR positive for *mcr-1* and confirmed as phenotypically colistin resistant. In contrast, a study in Argentina reported that 49% (n = 304) of *E*. *coli* isolates recovered from broilers were identified as colistin resistant by microdilution [59]. Another study in Brazil reported that 19.5% of chicken meat samples (n = 41) were positive for *mcr*-1 [60]. Colistin resistant Enterobacteriaceae have been described in humans and poultry in several Latin American countries [61,62]. Considering these findings and because colistin has been largely used as a growth promotor in Latin America, the poultry industry could be considered an important hotspot for this kind of resistance. Additionally, it has to be considered that although we did not find *mcr-2* in our study, up to 8 genetic determinants for colistin resistance have been described [63–65]. Therefore, a search for more genetic determinants along with a phenotypic screening assay should be conducted to better understand the epidemiology of colistin resistance in poultry from Ecuador.

Genes of the *bla*_CTX-M_ family have been the most prevalent ones in poultry production, even when there is no selective pressure due to antibiotic usage [47,66]. In our study, *bla*_CTX-M-65_ was the most prevalent allele of the *bla*_CTX-M_ family (33,6% of the ESBL-producing isolates) followed by *bla*_CTX-M-3_ (19,1%) and *bla*_CTX-M-55_ (17,3%) which differs from the results of other countries in the region. Colombia reported *bla*_CTX-M-2_ as the most prevalent variant followed by *bla*_CTX-M-8_ and *bla*_CTX-M-15_ [41], while Brazil identified *bla*_CTX-M-8_ and *bla*_CTX-M-2_ variants in chicken meat [40,42,67] In our case, *bla*_CTX-M-8_ and *bla*_CTX-M-2_ were present as a small proportion. Other genes such as *bla*_SHV-5_, *bla*_SHV-153_ and *bla*_TEM-176_ were found in lower proportions which agrees with the mentioned studies.

In Ecuador, there are no data about *bla*_CTX-M-65_ in *E*. *coli* from poultry. However, this variant has been identified in *Salmonella* from poultry and in human clinical samples in Ecuador [68]. Likewise, *bla*_CTX-M-3_ and *bla*_CTX-M-55_ have been identified in human infections [36,69]. These findings suggest the presence of plasmids carrying these variants in our environment. Therefore, transmission of resistance determinants from poultry to human may occur, but further evidence is needed to confirm this hypothesis. Finally, 6 isolates did not present ESBL or AmpC genes. In these cases, a broader panel of beta-lactamases genes should be used to identify the genetic determinants of resistance in these isolates.

Cross-contamination between farms could explain our finding that the same ESBL and AmpC genotypes originated from more than one farm. This idea is supported by other studies that found that *Salmonella* and *Campylobacter* isolated from different poultry farms are clonally related [70,71]. In Ecuador, climatic and social factors lead to most poultry houses having an open configuration in which implementation of rigorous biosecurity is difficult. Spread of bacterial genotypes among farms and integrated companies can therefore be a common event [72,73]. This highlights the importance of implementing effective biosecurity systems aiming not only to avoid the spread of AR but also to improve poultry health. Additionally, the contribution of factors like contamination of one-day-old chicks or feed should be studied in the future to obtain more insights on clonal relatedness of AR bacteria. It must be considered that, despite the relatively high concentration of cefotaxime used for screening of ESBL/AmpC phenotypes, a large number of isolates were recuperated.

In conclusion, poultry production systems represent a hotspot for AR in Ecuador, possibly mediated by the extensive use of antibiotics in this industry. Monitoring this sector in national and regional plans of AR surveillance should therefore be considered.

## Supporting information

S1 Fig REP-PCR profiles of the 176 tested Escherichia coli isolates. (PDF)
