# Supplementary figures and images for: Characterization of cefotaxime resistant Escherichia coli isolated from broiler farms in Ecuador
Source: PLoS One. 2019 Apr 5;14(4):e0207567. doi: 10.1371/journal.pone.0207567 (PMC6450624; doi:10.1371/journal.pone.0207567)

Figure 1. REP-PCR profiles of the 176 tested *Escherichia coli* isolates.

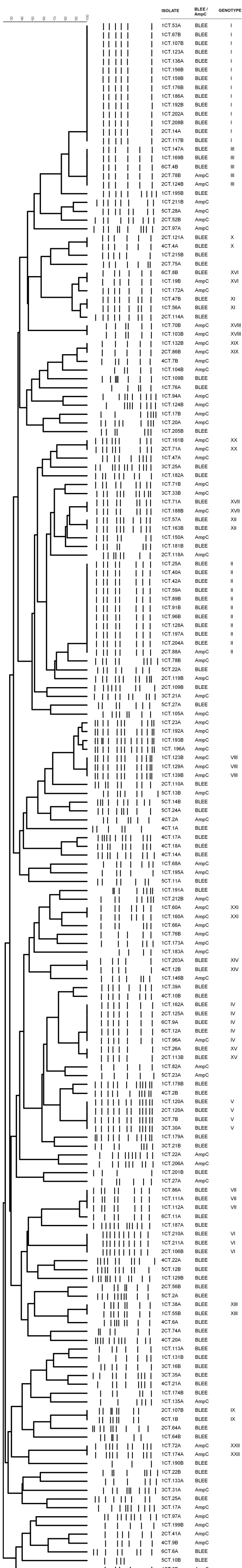

Supplement: S1 Fig — (PDF) [file pone.0207567.s001.pdf]
